# Application of X-ray computed tomography to analyze the structure of sorghum grain

**DOI:** 10.1186/s13007-022-00837-7

**Published:** 2022-01-11

**Authors:** Daniel Crozier, Oscar Riera-Lizarazu, William L. Rooney

**Affiliations:** 1 Department of Soil and Crop Sciences, Texas A&M University, College Station, TX 77843 USA; 2 Department of Horticultural Sciences, Texas A&M University, College Station, TX 77843 USA

**Keywords:** Grain quality, Grain morphology, Phenotyping, Segmentation, Machine learning, Random forest

## Abstract

**Background:**

The structural characteristics of whole sorghum kernels are known to affect end-use quality, but traditional evaluation of this structure is two-dimensional (i.e., cross section of a kernel). Current technology offers the potential to consider three-dimensional structural characteristics of grain. X-ray computed tomography (CT) presents one such opportunity to nondestructively extract quantitative data from grain caryopses which can then be related to end-use quality.

**Results:**

Phenotypic measurements were extracted from CT scans of grain sorghum caryopses. Extensive phenotypic variation was found for embryo volume, endosperm hardness, endosperm texture, endosperm volume, pericarp volume, and kernel volume. CT derived estimates were strongly correlated with ground truth measurements enabling the identification of genotypes with superior structural characteristics.

**Conclusions:**

Presented herein is a phenotyping pipeline developed to quantify three-dimensional structural characteristics from grain sorghum caryopses which increases the throughput efficiency of previously difficult to measure traits. Adaptation of this workflow to other small-seeded crops is possible providing new and unique opportunities for scientists to study grain in a nondestructive manner which will ultimately lead to improvements end-use quality.

**Supplementary Information:**

The online version contains supplementary material available at 10.1186/s13007-022-00837-7.

## Background

In sorghum [*Sorghum bicolor* (L.) Moench], breeders have primarily focused on yield improvement and stability; grain quality has been a trait of secondary importance. However, the demand for cereal grains that increase animal feed efficiency, address global malnutrition, improve food quality for human consumption, and meet niche-markets demands dictate the necessity for improvements in grain quality [1–3]. While breeders can select for improved grain quality, they must maintain grain yield and yield stability as any reduction would be detrimental to adoption and further reduce rates of genetic gain for yield. In wheat, it is possible to improve grain quality parameters without sacrificing agronomic performance, and the same may be true in sorghum [4].

A sorghum caryopsis is composed of three biological components: pericarp, endosperm, and embryo [5]. The relative size of each component varies among genotypes and production environment but pericarp, endosperm, and embryo account for around 7%, 84%, and 9% of kernel weight, respectively [6]. The pericarp is the outermost layers of a kernel and includes the epicarp, mesocarp, and endocarp layers. The thickness of the pericarp is associated with multiple traits which affect sensitivity to grain weathering, processing qualities (i.e., decortication), and storage stability [7, 8]. The endosperm is composed of protein and starch and is subdivided into the aleurone layer, peripheral, hard (vitreous), and soft (floury) portions [9]. Lastly, the embryo is composed of the embryonic axis and scutellum and contains protein and the majority of lipids, vitamins, and minerals found in the caryopses [6].

Studies on kernel structure in sorghum have traditionally involved hand dissection or sectioning of kernels followed by observing a single longitudinal cross sections [8, 10–13]. These methods destroy the seed, offer only one cross section (typically in the middle of the kernel), and are time consuming, which limits the number of kernels that can be evaluated per sample. Because kernel components vary across cross sections [5], cutting kernels in half is not representative of the whole seed resulting in biased estimates of kernel structure.

In sorghum, grain quality often depends on matching the end-use with physical kernel characteristics. Physical properties that affect grain quality include seed size, seed weight, endosperm texture, bulk density, and grain hardness [14]. Endosperm texture is the relative proportion of hard endosperm to soft endosperm [5]. As the name implies, hard (or vitreous) endosperm in sorghum is denser and more translucent than the soft endosperm, which is opaque and more porous. Endosperm texture is an important factor in the milling quality of grains and resulting flour, as well as susceptibility to grain mold [5, 12]. Generally, kernels with a higher portion of hard endosperm are preferred for milling because they are more resistant to breakage during decortication and yield cleaner endosperm of larger particle size giving a higher milling yield [5]. Because endosperm texture is difficult to measure, relatively few studies have examined the genetics controlling this trait [13]. Therefore, identification of better phenotyping methods may lead to gene discovery, improved selection efficiency, and advances in grain quality.

Computed tomography (CT) imaging technology is a powerful tool that can be utilized to measure complex features in biological specimens. The CT technology works by beaming x-rays through an object while rotating around the object in a helical path. The resultant x-ray signals are then processed using mathematical algorithms and stitched together into cross sectional images, or “slices” that are stacked together forming a three-dimensional image. From CT images, volumetric data can be analyzed for various structures with different densities. Until recently, the scale, resolution, throughput, accessibility and cost of this technology limited its use [15]. However, recent studies have demonstrated the increased through put and access ability of and accessibility of this technology to plant scientists [16, 17].

The advantages of CT imaging include nondestructive data acquisition, increased throughput and efficiency for gathering multiple traits, and more accurate measurements [15, 18]. Plant stems, leaves and roots of numerous plant species have been characterized by CT imaging [15–20]. In sorghum, Gomez et al. [16] developed a high throughput phenotyping system for morpho-anatomical stem properties for application in a crop improvement program. Therefore, it may be possible to develop similar methodology for analysis of grain although caryopsis present different challenges.

CT imaging has been used to analyze caryopses in other cereal grain crops. In rice, CT imaging distinguished high-amylose from wild-type rice [21]. In wheat, CT imaging assessed the damage caused by sprouting and insect infestation [22]. Similarly in corn, CT imaging effectively assessed damage from insect feeding and estimated kernel hardness [23, 24]. To estimate kernel hardness in corn, CT imaging was used to exclude regions not of interest (cavities and germ) to truly determine volumes and densities of the endosperm [23]. If similar methodology could be developed for quantifying traits in sorghum grain, it should be possible to assess sorghum endosperm texture on a three-dimensional basis for the first time. In addition, it may be possible to extract information on other traits such as the spatial distribution of endosperm, endosperm hardness, embryo size, kernel size, pericarp thickness, and identification of waxy endosperm.

CT scans produce a vast volume of images that requires efficient methods of data management and analytics. To extract information from CT scans, the images need to be simplified and partitioned into regions of interest. This process, segmentation, assigns a label to every pixel in an image based on certain common characteristics. From this, quantitative data can be extracted in the form of size and shape of objects in proportion to one another. The simplest approach to segmenting an image is to use thresholds based on pixel intensity to subdivide an image into different regions. However, there are limitations to this approach when sufficient differences in pixel intensity are lacking as is common in real world applications. Guelpa et al. [23] reported from CT scans of corn kernels that the density of the germ and hard endosperm were very similar and accurate discrimination between the two was not possible using thresholds based on pixel intensity. Other methods of segmentation include looking for acute changes in pixel intensity (edge detection), or changes in texture.

Machine learning approaches offer the potential to combine a collection of feature selection tools for improved image segmentation. There are numerous machine learning algorithms which have been applied to segment medical images and the varying strengths and weaknesses of many were reviewed by Seo et al. [25]. Random forests are easy to use, have fewer hyperparameters to tune than other models, and produce reduce results with high accuracy and stability [25]. The random forest classification method combines random uncorrelated decision trees into one prediction model where a decision tree is essentially a series of yes/no questions that lead to a predicted class [26]. The trees are trained on different sets of data and use different features to protect each other from their individual errors. In this classification scheme, some individual trees may be wrong, but most trees will be correct. Applying machine learning based approaches to segmenting CT images may alleviate the challenges put forth by Guelpa et al. [23] and prevent the use of manual image cleaning.

One of the challenges in implementing machine learning to a broader range of applications is the knowledge gap between scientists versed in machine learning and applied researchers. Trainable Weka Segmentation is a Fiji plugin that combines a collection of machine learning algorithms with a graphical user interface for ease of accessibility and functionality [27]. This software is freely available and can help bridge the gap between the machine learning and image processing fields.

Given limitations in accurate and nondestructive analysis of grain samples which impede improvements in sorghum grain quality, a phenotyping platform for CT imaging sorghum grain was developed. Thereafter, a diversity panel of sorghum was used to validate the effectiveness of CT imaging to measure structural characteristics in sorghum kernels.

## Materials and methods

### Plant material

A panel of 19 sorghum inbred lines representing a range of grain composition was used (Table 1). These lines varied for kernel traits including pericarp color, mesocarp thickness, presence or absence of the testa layer, kernel size, kernel hardness, and endosperm texture. Grain was bulk harvested from ten panicles for each line in 2019 at physiological maturity in College Station, Texas and stored in a cold vault at 11–13% moisture until scans were conducted.

**Table 1:** Plant material and phenotypic kernel characteristics of 19 sorghum inbred lines evaluated in this study

Genotype	Pericarp Color	Mesocarp Thickness	Testa Layer Presence	References
Ajabsido	White	Thick	Yes	[28]
BOK11	White	Thick	No	[29]
BTx2928	White	Thick	No	[30]
BTx378	Red	Thick	No	[31]
BTx399	Red	Thick	No	[31]
BTx642	Yellow	Thick	No	[32]
BTxArg-1	White	Thin	No	[33]
Dorado	White	Thin	No	[34]
FC6601_Spur Feterita	White	Thick	Yes	[35]
ICSV400	White	Thin	No	[36]
ICSV745	White	Thin	No	[37]
RTx2536	White	Thin	No	Rosenow, unpublished data, (1964)
RTx430	White	Thin	No	[38]
SC103-12E (IS12170C)	Red	Thin	Yes	Rosenow, unpublished data, (1970)
SC283 (IS7173C-TAM)	White	Thin	No	Rosenow, unpublished data, (1972)
Standard Early Hegari (SN106)	White	Thick	Yes	[39]
Sureno	White	Thin	No	[40]
TAM2566	Red	Thin	Yes	[41]
Texas Blackhull Kafir (SN59)	White	Thick	No	[31]

### Experimental details

The experimental design was a randomized complete block design with three replications. An experimental unit, compromised of 40 sorghum kernels of a genotype, was placed in a single well in an expanded polystyrene foam microtube storage box. In total, each CT scan contained 21 experimental units constituted by 19 different genotypes. One entry (RTx430) was replicated three times to assess the extent of spatial variation within a scan. Three separate CT scans were completed; each scan was considered a replication.

### CT scanning and image processing

**Fig. 1:**
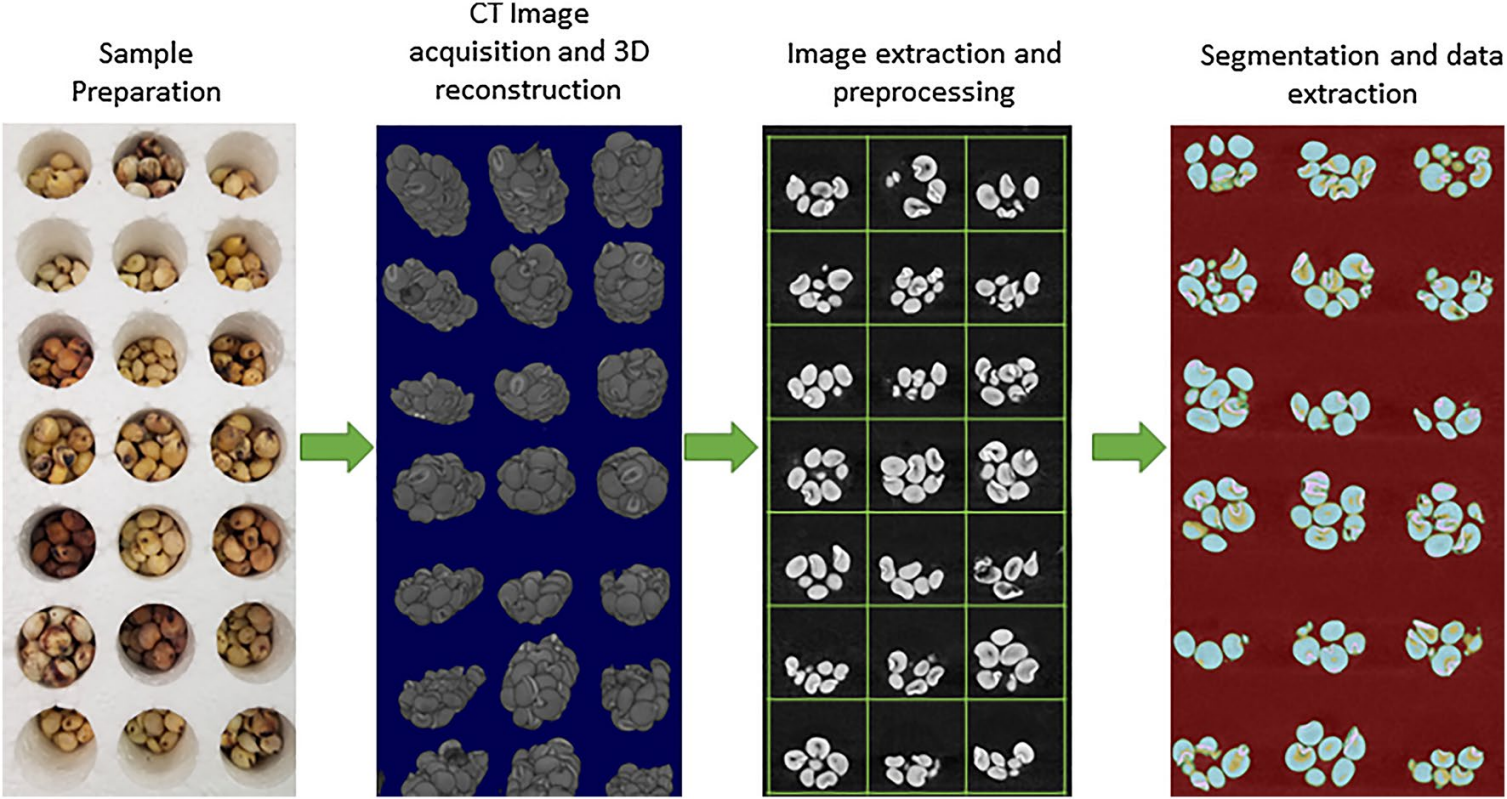
Overview of the CT imaging and data acquisition pipeline

The CT scans were performed by a North Star Imaging X50 industrial 3D X-ray inspection system. VorteX automated single pass computed tomography scanning technique, which utilizes spiral acquisition and reconstruction with a digital flat-panel detector, was used to construct images. This alleviated the use of volume stitching and provided uniform resolution in axial and sagittal slices across the entire volume. Scans took approximately 2.5 h to complete and provided a resolution of 20.2 µm. After imaging, 2-dimensional slices from the X-axis were exported as PNG files. From each scan, approximately 1500 images (~ 40 GB when uncompressed), were extracted. Stacks of two-dimensional slices were imported into Fiji [42] where they were converted to 8-bit grayscale to reduce file sizes. Stacks of images were then processed using the enhanced contrast feature with saturated pixels set to 0.3% and normalized using the stack histogram. This processing made manual identification of regions easier for use in training the segmentation classifier. An overview of this CT imaging and data acquisition pipeline can be visualized in Fig. 1, with further detail provided in Additional file 1.

### Image data extraction

A machine learning based plugin in Fiji, Trainable Weka Segmentation 3D [27], was used to segment stacks of images into different regions. A training set was built that was comprised of ten sequential images from each of the three scans where 52,966 pixels where manually annotated as belonging to one of five classes: background, pericarp, embryo, soft endosperm, or hard endosperm. Manual annotations were made such that there was representation among all genotypes, images, and scans in the training set. A classifier was then built using a fast random forest, a multithreaded version of the random forest put forth by Breiman [26], with 200 trees and two random features per node. In previous research it has been well documented that that classification accuracy does not increase if using more that 100–200 trees with most data sets, while processing time increases linearly with the addition of more trees [43–45]. Within this data set, increasing tree number from 200 to 400 increased training time by 111% while only increasing pixel classification by 0.01%. Therefore, since random forests produce a limiting value of the generalization error but do not overfit as more trees are added, 200 trees were used as a compromise between computational power and the diminishing returns in performance gain with more trees [26].

Three training features were used: Hessian, mean, and variance with a minimum sigma of one and a maximum sigma of eight. Mean and variance are texture-based filters useful in differentiating between areas that do not have distinct boundaries but contain patterns of homogenous variation. In the present study, this was selected because it was useful in delimiting between the regions of sorghum kernels where there was a gradation in pixel values as opposed to distinct boundaries. Hessian is an edge detection filter useful in discriminating the borders of objects defined by clear boundaries such as that between the caryopsis and background. The random forest model described above was evaluated within the training set using ten iterations of fivefold cross validation and compared against three other models to determine relative accuracy and computational efficiency (Table 2). Hyperparameter tuning was done to optimize performance while keeping training times similar to the random forest model. A support vector classifier (SMO) was fit using a logistic calibrator, a polynomial kernel, a complexity of six and an epsilon value of 1.0E^−12^. The Naive Bayes model was fit using default parameters as few tuning options were available. A neural network (MultilayerPerceptron) model was fit with ten hidden layers, a learning rate of 0.3 with momentum 0.2, and a training time of 20. More information on these models can be found within the Weka user manual [27].

**Table 2:** Performance of machine learning classifiers in segmenting CT scans of sorghum grain

Classifier	Training time (s)	Root mean squared error	Percent Correct
Random Forest	3.5	0.03	99.9
SMO	3.4	0.32	98.1
Naive Bayes	0.2	0.15	93.7
Multilayer perceptron	3.6	0.07	98.2

Within the training set, the random forest model was not the fastest; however, it had the highest pixel classification accuracy of 99.9%, and lowest root mean squared error (Table 2). Although in depth follow up studies are necessary for optimization of classifier models to produce the best results and fairly compare between classifiers, the random forest classifier was deemed good enough and used for subsequent analysis.

File sizes in excess of 9 GB after initial processing were too large to segment at once. Thus, stacks of images were subdivided into 21 smaller tiles of more manageable size (~ 250 MB). Every genotype was allocated to an individual tile and segmented separately using the common classifier. Pixel counts for each of the five classes were obtained for every experimental unit and retained for further use.

Total pixel number of kernels for each experimental unit was calculated by adding the number of pixels containing hard endosperm, soft endosperm, pericarp and embryo. A reference point in the image was measured to find pixels per mm^2^. Average single kernel volume was then calculated by converting total pixel number to mm^3^ and dividing that by 40 (the numbers of kernels/per entry). Average embryo, endosperm, and pericarp volume were calculated similarly to average kernel volume using the pixel number for each respective region. Endosperm texture was calculated by dividing the total number of pixels containing hard endosperm, by the total number of pixels containing soft endosperm for each experimental unit.

Not using the segmentation classifier, average endosperm intensity of each experimental unit was calculated by averaging the range of pixel values in all 40 kernels across all slices. Pixel values range in brightness from 1 to 255, where higher pixel values are brighter and represent denser objects in a CT scan. Pixel values below 70 were ignored as background, and pixel values above 248 were ignored as embryo. This was done to achieve an approximation of endosperm hardness based on density, hence exclusion of background noise and regions containing pericarp or embryo.

### Ground-truth data collection for validation

Reference grain quality parameters for each genotype were established using both quantitative and visual subjective tests. First, the Single Kernel Characterization System (SKCS) (SKCS 4100, Perten Instruments North America Inc., Springfield, IL) was used to measure diameter, weight, and hardness of 300 individual kernels. This method is widely used in the wheat industry and accepted by the sorghum industry as a tool for measuring grain characteristics [46].

Visual assessment of endosperm texture was estimated by cutting three kernels from each genotype longitudinally along the embryo to bisect the caryopsis, and visually scoring them based on the ratio of hard to soft endosperm. Genotypes were placed into categories from one to five where one is greater than 80% soft endosperm, two is 80% to 60% soft endosperm, three is 60% to 40% soft endosperm, four is 40% to 20% soft endosperm, and five is less than 20% soft endosperm. This was done analogous to traditional phenotyping methods in which the reliability of data is subject to the skill and expertise of the scorer [47].

### Statistical analysis

Restricted maximum likelihood (REML) analysis was conducted in JMP (Version *15.0.0*. SAS Institute Inc., Cary, NC) using the model:

$$ Y_{ij} = \, u \, + \, Gen_{j} + \, Rep_{i} + \, Col_{k} + \, Row_{l} + \, E_{ij} $$

where *Y*_*ij*_ is the trait of interest, *u* is the mean effect, *Gen*_*j*_ is the effect of the *j*th genotype, *Rep*_*i*_ is the effect of the *i*th replicate, *Col*_*k*_ is the effect of the *k*th column, *Row*_*l*_ is the effect of the *l*th row, and *E*_*ij*_ is the random error term. Inclusion of spatial corrections, row and column, was done to assess and account for variance within CT scans. It was hypothesized that objects in the center of a CT scan may appear denser possibly due to changes in attenuation from the x-ray passing through more material, or if the imaging gantry does not travel far enough past the ends of the sample to record an accurate image. Factors with negative variance components were removed from the model. Normality of residuals from the models were checked using the Anderson–Darling test, and log base ten transformations were used to normalize non-normal traits. All random models were used to generate estimated best linear unbiased predictors (EBLUPs) for each genotype, variance components, and repeatability estimates. Repeatability (R) on an entry-mean basis was calculated using the equation:

$$ R \, = {{\left( {\sigma^{2}_{g} } \right)} \mathord{\left/ {\vphantom {{\left( {\sigma^{2}_{g} } \right)} {\left( {\sigma^{2}_{g} + \, \left( {{{\sigma^{2}_{e} } \mathord{\left/ {\vphantom {{\sigma^{2}_{e} } r}} \right. \kern-\nulldelimiterspace} r}} \right)} \right)}}} \right. \kern-\nulldelimiterspace} {\left( {\sigma^{2}_{g} + \, \left( {{{\sigma^{2}_{e} } \mathord{\left/ {\vphantom {{\sigma^{2}_{e} } r}} \right. \kern-\nulldelimiterspace} r}} \right)} \right)}} $$

where *R* = the repeatability, *σ*^*2*^_*g*_ = the genotypic variance, *σ*^*2*^_*e*_ = the error variance, and *r* = the number of replications. Repeatability, calculated similar to heritability, indicates the consistency of data and is used in the absence of family structure. Pearson correlation coefficients (*r*) were computed to assess the relationship between EBLUPs of CT-derived traits and validate against ground-truth data. Best linear unbiased estimators (BLUEs) were estimated using the aforementioned models with genotype being considered as a fixed effect and all other factors random effects. The Tukey – Kramer honestly significance difference (HSD) test was used to determine if genotypes were significantly different from one another using BLUEs.

## Results and discussion

### Phenotypic variation

**Fig. 2:**
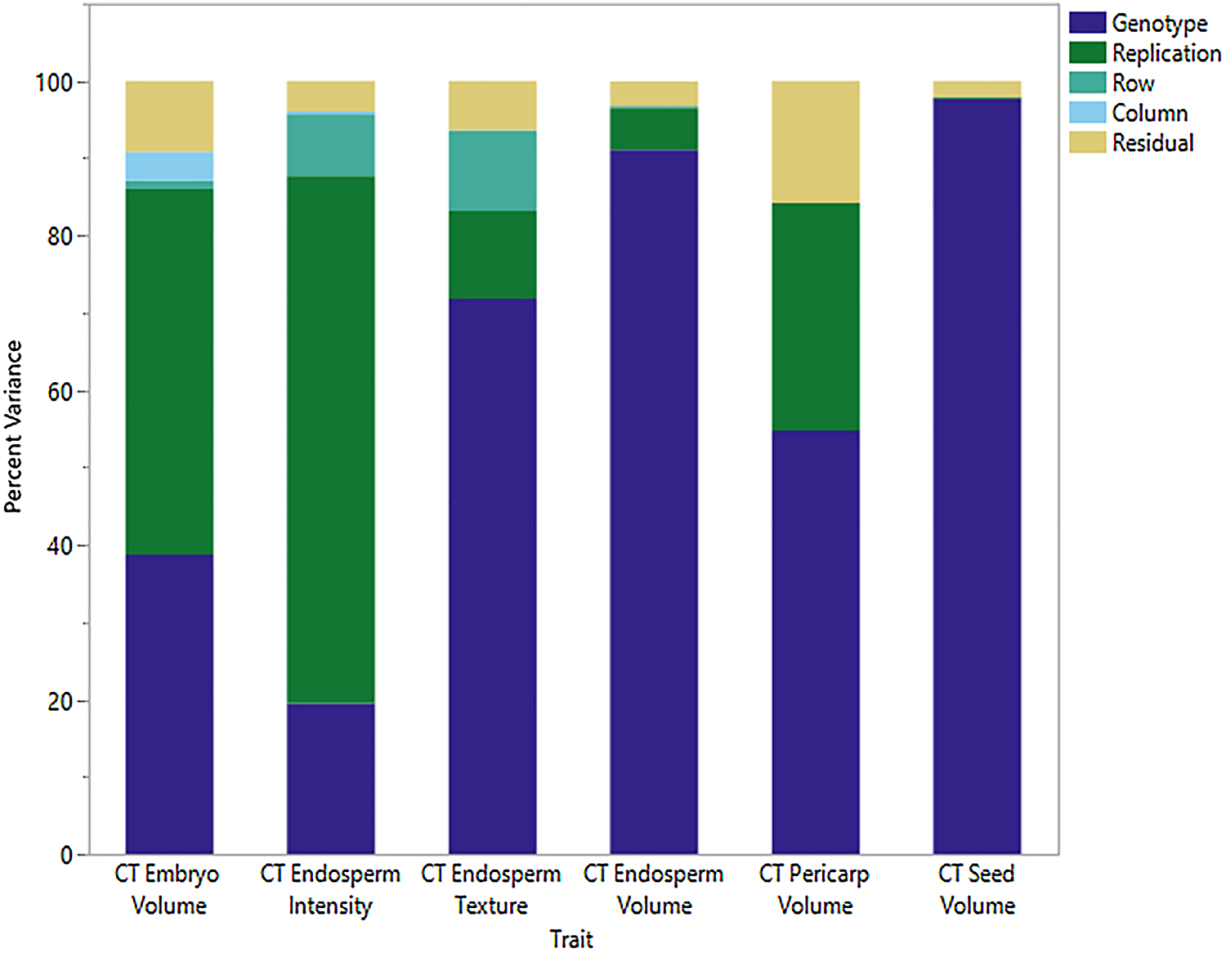
Percent variance associated with factors in CT-derived estimates of sorghum kernel structure for 19 genotypes. Replication refers to independent CT scans while row and column refer to the spatial position within the CT machine

Significant (*p* < 0.01) variation among genotypes was detected for embryo volume, endosperm intensity, endosperm texture, endosperm volume, pericarp volume, and kernel volume using CT imaging. Variance component decomposition shows genotype and rep were the largest sources of variation across traits (Fig. 2). Residual errors were small which resulted in high *R*^2^ values and repeatability (*R*) estimates for all traits (Table 3).

**Table 3:** Best linear unbiased estimators for CT-derived measures of sorghum grain structure for 19 sorghum genotypes

Genotype	CT seed size (mm^3^)	CT pericarp volume (mm^3^)	CT embryo volume (mm^3^)	CT endosperm volume (mm^3^)	CT endosperm texture	CT Endosperm Intensity
Ajabsido	29.12	2.21	4.05	22.77	1.79	181.59
BOK11	14.30	1.54	2.03	10.77	1.43	178.68
BTx2928	16.94	1.76	2.53	12.68	2.19	181.27
BTx378	20.56	2.03	2.81	15.56	1.69	179.17
BTx399	24.13	2.02	3.33	18.79	2.69	185.63
BTxArg-1	12.55	1.30	1.82	9.24	2.98	183.87
BTx642	18.13	1.76	2.46	13.65	3.51	186.49
Dorado	20.86	1.93	2.62	16.20	2.54	184.34
ICSV400	24.79	2.10	3.74	18.84	2.97	187.32
ICSV745	11.82	1.24	1.73	8.82	3.96	184.98
Tx2536	22.54	2.05	3.07	17.25	2.29	182.79
RTx430	27.00	2.26	4.14	20.46	2.31	182.86
SC103-12E	20.19	2.00	2.72	15.10	1.08	174.55
SC283	15.83	1.51	2.91	11.40	3.99	188.32
Spur Feterita (FC6601)	26.91	2.24	3.68	21.06	1.48	178.72
Standard Early Hegari (SN106)	19.40	1.70	2.54	14.91	1.41	176.68
Sureno	14.68	1.49	2.38	10.97	5.11	187.8
TAM2566	20.22	1.82	2.70	15.71	1.43	179.46
Texas Blackhull Kafir (SN59)	17.66	1.70	2.06	13.74	1.35	175.29
Average	19.87	1.82	2.81	15.15	2.43	182.10
HSD	2.15	0.49	1.36	2.39	1.53	6.14
*R*^2^	0.92	0.97	0.95	0.98	0.88	0.99
*R*	0.97	0.94	0.97	0.99	0.91	0.99

*HSD* honestly significant difference from Tukey-Kramers test, *R*^*2*^ = total variation explained by the model, *R* = repeatability

The variation associated with replication was variable (Fig. 2). Some traits, such as CT Endosperm Intensity, had a large replication effect which is likely due to subtle differences in average intensity values between scans (e.g., some scans were brighter than others). Therefore, if data is extracted from multiple CT scans, control genotypes or reference objects of known density are necessary to normalize all scans to the same range of intensity values. Traits derived using the machine learning classifier were less affected than endosperm intensity by replication effects because a combination of features (e.g., mean, variance and Hessian) was used as opposed to pixel intensity alone (Fig. 2).

Spatial variation, accounted for in the model by row and column position in CT scans, were not significant sources of variation for any traits but a small amount of variance was partitioned to spatial effects. Given the conservative approach used to assess significance of effects, spatial variation may still be present within scans and when so, it is likely due to changes in attenuation from the x-ray passing through more material, or if the imaging gantry did not travel far enough past the ends of the sample to record an accurate image.

Across all genotypes, sorghum kernel composition measured using CT imaging averaged 9% pericarp, 76% endosperm, and 14% embryo by volume (Table 3). Previous literature reported kernel composition ranges as 4.3–8.7% for pericarp, 81.7- 86.5% for endosperm, and 8–10.9% for embryo by weight [6], so CT-estimates are slightly higher for pericarp and embryo with a concomitant reduction in endosperm proportion. These differences could be due to several factors including the different densities of tissues, genotypes sampled, environmental effects, and measuring methodology. Average kernel volume determined by CT imaging was 19.9 mm^3^; the most recent U.S. Grains Council survey reported similar sorghum kernel volumes of 19.3 mm^3^ in 2015 and 20.6 mm^3^ in 2016 in the United States [48].

### Correlations and validation

**Fig. 3:**
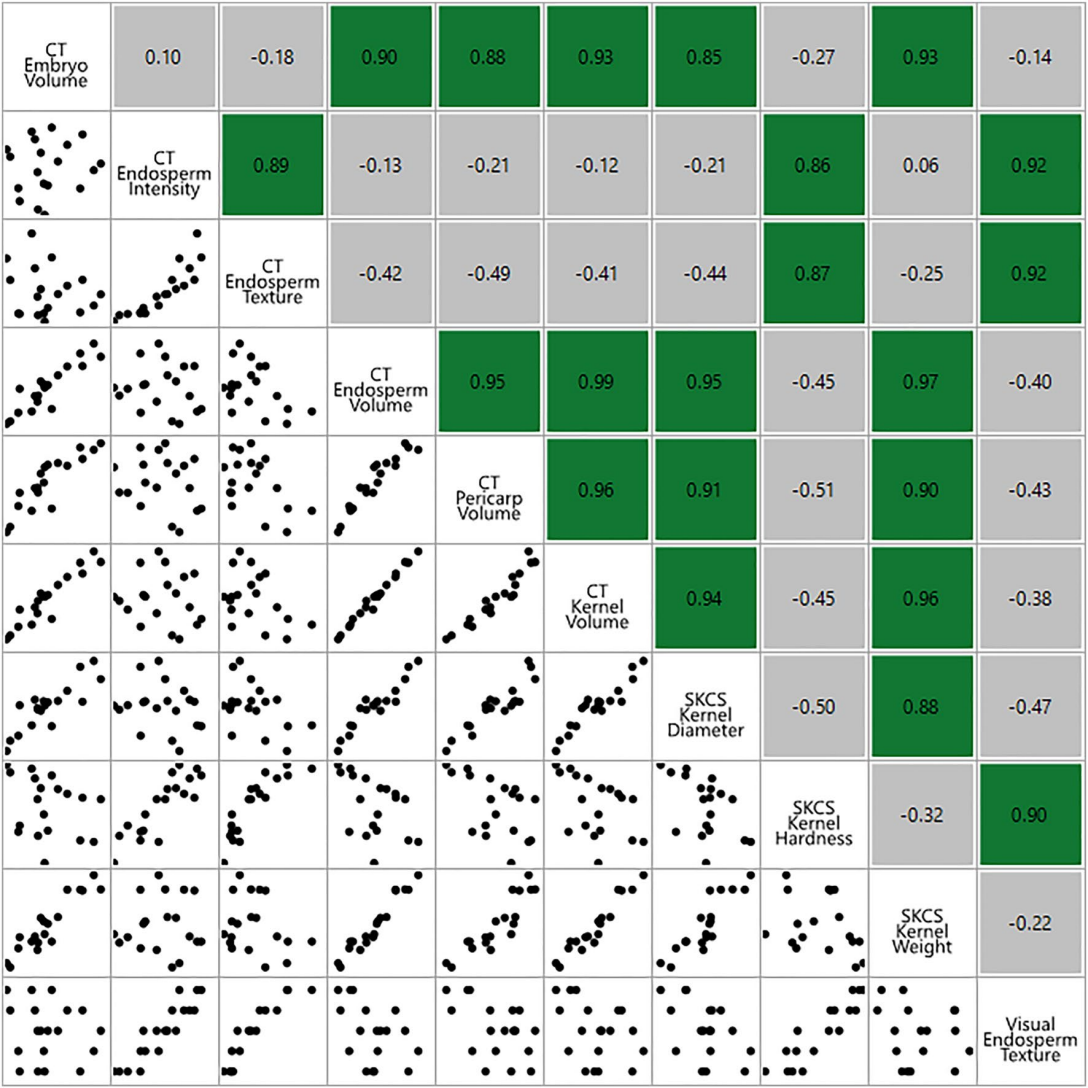
Correlations among different sorghum structural characteristics as measured by CT imaging, SKCS, and visual scoring. Pearson’s correlation coefficients significant at *p* < 0.01 are colored green and shown in the top right. Graphic depictions of the correlation scatterplot matrices are presented in the lower left

Strong correlations were observed between many CT-derived trait measurements (Fig. 3). As expected, CT kernel volume was correlated (*p* < 0.01) with CT embryo volume, CT endosperm volume, and CT pericarp volume because larger grains are naturally comprised of greater volumes of embryo, endosperm, and pericarp. CT endosperm intensity was correlated (*p* < 0.01) with CT endosperm texture, which is logical given the differences in density between soft and hard endosperm [5].

Estimates of CT-derived traits were also correlated with ground-truth measurements from SKCS and visual scoring. Visual scoring for endosperm texture was highly correlated (*p* < 0.01) with both CT endosperm intensity and CT endosperm texture (Fig. 3). SKCS kernel hardness was also strongly correlated (*p* < 0.01) with both CT endosperm intensity and CT endosperm texture (Fig. 3). SKCS kernel diameter and SKCS kernel weight were both correlated (*p* < 0.01) with CT kernel volume (Fig. 3). The strong correlations reported herein suggest that CT-derived trait measures are reliable.

### CT imaging challenges

**Fig. 4:**
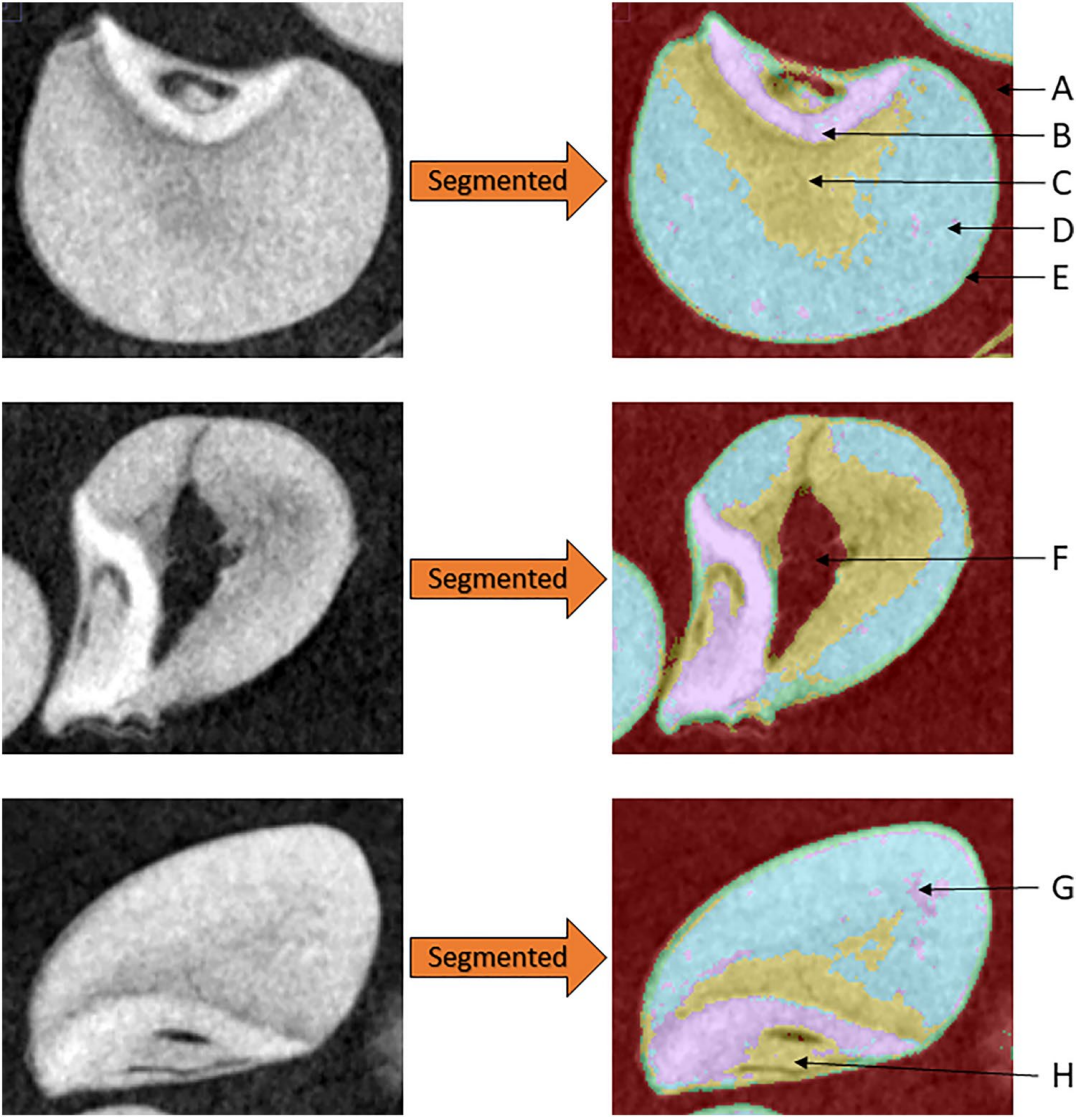
Images of sorghum kernels before and after segmentation. **A** background, **B** embryo, **C** soft endosperm, **D** hard endosperm, **E** pericarp, **F** hollow void in kernel, **G** hard endosperm misclassified as embryo, **H** less dense embryonic axis region of embryo misclassified as soft endosperm

CT images were segmented into regions containing background, embryo, soft endosperm, hard endosperm, and pericarp (Fig. 4). In addition, other phenotypic kernel characteristics, such as the presence of cracks and voids, were observed within some genotypes (Fig. 4). Since the segmentation classifier used herein detected these large hollow voids and removed them from kernels, the estimates of sorghum kernel structure remained unbiased.

Histograms of pixel values for scanned grain lacked distinct peaks and valleys corresponding to individual regions of the caryopsis like that reported by Guelpa et al. [23] in corn. This made segmentation more difficult and necessitated the use of a more complex approach than relying singularly on intensity value of regions. Some errors in classification were present, likely due to the lack of marked differences between regions of the kernel. For example, regions of hard endosperm were occasionally misclassified as embryo and regions of the embryo were occasionally misclassified as soft endosperm (Fig. 4). This is because even within regions, pixel intensity and texture are not homogenous. For instance, within the embryo, the scutellum is denser and therefore brighter than the embryonic axis. This could lead to higher residual errors in models and either over or underestimation of some regions of the kernel. Potential ways of accounting for this in future analysis would involve thorough testing and optimization of training set size, number of trees in random forest models, features, sigma values, and other machine learning approaches. However, future evaluations should balance model accuracy with computational efficiency as to not slow throughput efficiency of subsequent phenotypic analysis. Overall, misclassifications were minimal and did not negatively impact data quality, but they may explain why embryo and pericarp volume estimates were slightly higher than previously reported.

### Genotypic differences

With the methodology presented herein, structural characteristics of sorghum caryopsis can be quantified. This serves many potential applications to plant breeders and cereal chemists alike for use in gene discovery, physiological studies, and other research. One such application is to discriminate between genotypes. Significant genotypic differences were detected between genotypes for all traits using CT imaging (Table 3). For a trait like endosperm texture, CT imaging detected quantitative genotypic differences with more statistical differences between genotypes than categorical visual scoring (Table 3). In addition, genotypes can be selected for different end-use purposes based on structural characteristics. For milling, a genotype (such as BTx399) which has larger kernels, higher percent endosperm, and harder endosperm is preferred (Table 3). Also, cracks and voids which were observed in some genotypes (Ajabsido) are undesirable for milling as kernels would be more prone to breakage during harvest and decortication.

Among the 19 sorghum lines evaluated herein was one with waxy endosperm (BTxARG-1). Waxy endosperm is caused by a genetic mutation that inhibits the synthesis amylose resulting in a glossy endosperm phenotype that is slightly less dense and phenotypically distinct from normal endosperm [5, 49]. Efforts to discern between waxy and regular endosperm using CT imaging were not successful. Consequently, the approaches used to measure endosperm properties characterized material similarly regardless of endosperm type. BTxARG-1 (waxy), was classified as around the same relative ranking for hardness and texture by SKCS, visual, and CT scanning. Therefore, there is no evidence to suggest separate phenotyping methods are needed for waxy and non-waxy genotypes using the phenotyping pipeline provided.

## Conclusions

The phenotyping pipeline presented herein can be automated using the source code in Fiji and did not require manual input past training the initial classifier; thus, increasing throughput efficiency of previously difficult to measure traits. This allowed accurate classification of endosperm texture as well other sorghum kernel structural characteristics. Based on the results presented herein, CT imaging presents new and unique opportunities for scientists to study sorghum grain in a nondestructive manner. With the capability to three-dimensionally segment sorghum kernels into regions, future studies can assess the spatial distribution and relationship structural characteristics have on other grain quality traits.

## Supplementary Information

**Additional file 1: Fig. S1.** Overview of data extraction from CT images of sorghum caryopsis.

## Data Availability

The datasets used and/or analyzed during the current study are available from the corresponding author on reasonable request.

## Abbreviations

BLUEs: Best linear unbiased estimators
CT: X-ray computed tomography
EBLUPs: Estimated best linear unbiased predictors
HSD: Honestly significance difference
R: Repeatability
REML: Restricted maximum likelihood
SKCS: Single Kernel Characterization System
